# Ras-like family small GTPases genes in *Nilaparvata lugens*: Identification, phylogenetic analysis, gene expression and function in nymphal development

**DOI:** 10.1371/journal.pone.0172701

**Published:** 2017-02-27

**Authors:** Weixia Wang, Kailong Li, Pinjun Wan, Fengxiang Lai, Qiang Fu, Tingheng Zhu

**Affiliations:** 1 State Key Laboratory of Rice Biology, China National Rice Research Institute, Hangzhou, China; 2 College of Biological and Environmental Engineering, Zhejiang University of Technology, Hangzhou, Zhejiang, P.R. China; Institute of Plant Physiology and Ecology Shanghai Institutes for Biological Sciences, CHINA

## Abstract

Twenty-nine cDNAs encoding Ras-like family small GTPases (RSGs) were cloned and sequenced from *Nilaparvata lugens*. Twenty-eight proteins are described here: 3 from Rho, 2 from Ras, 9 from Arf and 14 from Rabs. These RSGs from *N*.*lugens* have five conserved G-loop motifs and displayed a higher degree of sequence conservation with orthologues from insects. RT-qPCR analysis revealed *NlRSGs* expressed at all life stages and the highest expression was observed in hemolymph, gut or wing for most of *NlRSGs*. RNAi demonstrated that eighteen *NlRSGs* play a crucial role in nymphal development. Nymphs with silenced *NlRSGs* failed to molt, eclosion or development arrest. The qRT-PCR analysis verified the correlation between mortality and the down-regulation of the target genes. The expression level of nuclear receptors, Kr-h1, Hr3, FTZ-F1 and E93 involved in 20E and JH signal pathway was impacted in nymphs with silenced twelve *NlRSGs* individually. The expression of two halloween genes, *Cyp314a1* and *Cyp315a1* involved in ecdysone synthesis, decreased in nymphs with silenced *NlSar1* or *NlArf1*. *Cyp307a1* increased in nymphs with silenced *NlArf6*. In *N*.*lugens* with silenced *NlSRβ*, *NlSar1* and *NlRab2* at 9^th^ day individually, 0.0% eclosion rate and almost 100.0% mortality was demonstrated. Further analysis showed *NlSRβ* could be served as a candidate target for dsRNA-based pesticides for *N*.*lugens* control.

## Introduction

The rice brown planthopper (*Nilaparvata lugens* (Stål) (Hemiptera: Delphacidae)) is one of the most destructive monophagous insect pest of rice, *Oryza sativa*, it draw nutrients from the phloem of rice plants and cause huge yield losses every year in rice grown throughout tropical, subtropical and temperate areas in Asia[1,2]. RNAi (targeted silencing of gene expression by injection of corresponding dsRNA) has become a major tool in functional analysis of genes in insects [3, 4]. A decade of research on RNAi in insects has demonstrated that dsRNA could be developed as tailor-made pesticides for its sequence specificity and ability to suppress genes critical for insect survival [5]. *N*.*lugens* has a robust RNAi response to dsRNAs [6]. This offers great opportunities to analyze gene function by RNAi and search target for dsRNA-based pesticides in *N*.*lugens*.

Ras-like family small GTPases (RSGs) are components of signaling pathways that link extracellular signals via trans-membrane receptors to cytoplasm and touch on virtually every aspect of cell biology, including growth, differentiation, morphogenesis, cell division and motility, cytokinesis, and trafficking through the golgi apparatus, nucleus, and endosomes [7–9]. RSGs share a conserved structure and biochemical properties, acting as binary molecular switches turned on by binding GTP and off by hydrolyzing GTP to GDP. They are grouped into five major subfamilies Ras, Rab, Rho, Ran and Arf based on their sequence and functional similarities [10, 11]. Members from different subfamilies share a maximum of 40% identity. The functional diversity of these proteins is based on minor modifications in sequence, structure, and/or regulatory posttranslational modifications [12]. Moreover, members of the same family differ from each other in their “variable” membrane targeting domains, which dictate subcellular localization and dynamic spatiotemporal regulation. Conversely, distinct members of the RSGs transact and interconnect to each other through complex signaling networks [13]. Ras genes were first identified and characterized as transduced oncogenes in human tumor cells, Their dysfunction plays a crucial role in the pathogenesis of serious human diseases, including cancer and developmental syndromes[14, 15]. Since then, RSGs genes are described or predicted in diverse insect species, mostly based on sequence similarity, and demonstrated to participate in sorting and amplification of transmembrane signals, translocation of proteins, vesicular traffic, cell polarity, autophagy, cellular immune response etc [16–19]. In *Aedes aegypti*, Rheb GTPase was required for amino acid mediated activation of the TOR pathway that controls egg development. When Rheb was down-regulated, the egg development was severely hindered [20]. In Drosophila, Rheb is required for both cell growth (increase in mass) and cell cycle progression and the effects of Rheb are mediated by TOR [21, 22]. Arl1 is required for normal wing development and formation of secretory granules [23]. Rac2 is required for normal feeding and mating behaviour [24]. Rho GTPase signaling in *Apis mellifera* mushroom body medidated foraging-dependent growth [25]. Rab4b takes part in metamorphosis by regulating gene transcription and glycogen level in the insulin and 20E signal pathways in *Helicoverpa armigera* [26]. Knock down of Ran by RNAi resulted in the suppression of 20E regulated genes including EcR-B1, USP1, E75B, BR-CZ2, and HR3 [27]. These results suggest that RSGs involved in fundamental developmental process and showed functional diversity in insects. However, almost all the researches of RSGs focused on homometabolous insect development, their functions in hemimetabolous are still unknown.

In this study, twenty-nine RSGs genes were cloned and characterized in hemimetabolous insect *N*.*lugens*. The patterns of gene expression in different tissues and at different developmental stages were examined. Furthermore, the importance of each RSGs gene in *N*.*lugens* development was studied by RNAi. To our knowledge, this is the first report to describe and functionally analyze RSGs in *N*.*lugens*. Our results could provide basic information for understanding RSGs function in *N*.*lugens*.

## Materials and methods

### Insects rearing and samplings

*N*.*lugens* used in this study were originally collected from rice field in China National Rice Research Institute, Hangzhou, China, in 2008. The insects were reared on rice variety Taichung Native1 (TN1, a *N*.*lugens* -susceptible rice cultivar) in nylon mesh cages under conditions(28°C, 85% relative humidity and 16 h light/8 h darkness). For temporal expression analysis, the first- through fifth-instar nymphs and female, male adults were sampled respectively. Each sample contained 10 individuals and was repeated in biological triplicate. The tissues including salivary gland, gut, fat body, ovary, leg, wing, integument and hemolymph were dissected from 100 adult females, 2 days after eclosion, under a Leica S8AP0 stereomicroscope. Each sample was repeated in biological triplicate. The samples were frozen in liquid nitrogen and stored at -80°C until use. The third instar (one-day old) nymphs were used for dsRNA treatment and subsequent analysis.

### RNA extraction and qRT- PCR analysis

Total RNAs were isolated from whole bodies of the first- through fifth-instar nymphs, adults and from tissues or the nymph survivors of the dsRNA bioassay with RNeasy mini kit (Qiagen, Germany) according to manufacturer’s instruction. The potential genomic DNA contamination was removed by a treatment with DNase I kit (Qiagen Germany) after the RNA extraction. RNA concentration and quality was determined using a Nanodrop spectrophotometer (Thermo Scientific, USA). The first-strand cDNA were synthesized by reverse transcriptase using ReverTra AceqPCR RT Kit (ToYoBo, Osaka, Japan). The 10-fold diluted first-strand cDNA (2.0μL) was used as template for qRT-PCR.

mRNA abundance of each gene was tested in three technical replicates for each of three biological replicates. qRT-PCR were performed using a qPCR master mix SYBR^®^ Premix (Toyobo, Osaka, Japan) on ABI 7500 System (Applied Biosystems). The accompanying software was used for qPCR data normalization and quantification. Relative value for the expression level of target gene in development stages and tissues was calculated by the equation Y = 10 ^(Ct internal- Ct target)/3^ x100% [28], using the geometric mean of 18S rRNA (JN662398) and β-actin (EU179846) as internal [29]. Duncan's Multiple Comparison was used to determine differences among tissues and stages. Values of p<0.01 were considered significant.

### Identification, cloning and sequence analysis of RSGs genes

To identify the genes related to RSGs, we conducted sequence search in EST data (http://bphest.dna.affrc.go.jp/) based on molecular function and transcriptome database of *N*.*lugens* based on the functional annotation. Twenty-nine cDNA sequences in *N*.*lugens* similar to RSGs were obtained and assembled. The assembled sequences were used as queries to find homologs in the *N*.*lugens* genome (GenBank assembly GCA_000757685.1.) by BLAST search. The correctness of sequences with complete open reading frames (ORFs) was substantiated by common PCR techniques using the gene-specific primers. The gene-specific primers were designed using the Primer Premier 5.0 program based on the assembled sequences as shown in S1 Table. All the primers were synthesized by Invitrogen. Each PCR products specific to the target genes was individually cloned into the pCRII-TOPO vector (Invitrogen). The plasmids were extracted from several independent subclones with high pure plasmid isolation kit (Roche,Germany) and sequenced at Shanghai Boshang Ltd. Translations of cDNAs and predictions of the deduced proteins were conducted using DNAStar software (DNASTAR Inc., Madison, USA). Sequences were aligned using ClustalW and then a phylogenetic tree was generated using the neighbor joining method with MEGA 5.10 software (http://www.megasoftware.net/). Robustness of the branches was assessed by performing a bootstrap analysis of 1000 replications. Prediction of transmembrane helices and motif discovery in proteins were conducted using TMHMM Server v. 2.0 (http://www.cbs.dtu.dk/services/TMHMM/) and SMART (http://smart.embl-heidelberg.de/). Exon/intron organization of each Ras family gene was checked by aligning ORF with genomic DNA using the spidey program http://www.ncbi.nlm.nih.gov/spidey/spideyweb.cgi/ and GSDS online http://gsds.pku.edu.cn/.

### RNA interference and bioassay

dsRNAs of target genes and GFP were synthesized *in vitro* with PCR-generated DNA templates using the MEGAscript T7 Transcription Kit (Ambion, Austin, TX, USA). The sequence including full ORF regions were chosen for dsRNA synthesis for the twenty-eight genes. The specific primers used to generate these DNA templates are shown in S1 Table and a 23 base T7 promoter sequence (TAATACGACTCACTATAGGG) was added to the 5’end of each gene specific dsRNA synthesis primer. dsRNAs for each of twenty-eight genes were adjusted into the concentration of 700ng/μL and injected into the third instar (1-day old) nymph with 0.1μL according to previously described techniques [29, 30]. In parallel, *N*.*lugens* injected with GFP dsRNA or double distilled water were used as the negative control. After a 12-hour recovery period, the survived nymphs (at least 30 individuals) were selected and reared on 30- to 35-day-old plants of rice variety TN1 in one cage. Four days after injection, RNA was isolated from five survivors to test the efficiency of RNAi knockdown using qRT-PCR as described above. The mRNA levels of target gene, eight nuclear receptor genes *(NlFTZ-F1*, *NlKr-h1*, *NlECR*, *NlMet*, *NlBr-C*, *NlE75*, *NlHr3* and *NlE93*) and five Halloween genes (*NlCyp302a1*, *NlCyp306a1*, *NlCyp307a1*, *NlCyp315a1* and *NlCyp314a1*) were normalized to the endogenous reference gene 18S rRNA and actin. The relative amounts of gene transcripts were expressed as a ratio between treated group and control group by 2^-ΔΔCt^ method [31]. Three replicate injections were established in each of the dsRNA treatments and control. Duncan’s tests were used to determine difference between treatment and control. Values of p<0.05 were considered significant.

Mortality and development defects of dsRNA injected insects were recorded at 48 h time intervals until adult eclosion. Three independent biological replicates were carried out. To test for an effect of treatment, ANOVAs were performed using the percentage of survival nymphs or the cumulative percentage of eclosion as the dependent variable and treatment dsGFP injection, Duncan’s tests were used to determine differences among groups when treatment effects were detected.

## Results

### Identification of RSGs genes in *N*.*lugens* and sequence analysis

Based on the transcriptome and EST data, twenty-nine cDNA sequences encoding putative RSGs were assembled and amplified with PCR. BLAST analysis revealed that the putative amino acid sequence shows high homology to corresponding RSGs in other insects. We named these genes according to the names of their orthologues in *Zootermopsis nevadensis* or *Acyrthosiphon pisum*. The putative start codon was preceded by an in-frame stop codon in all this twenty-nine sequences. Among the twenty-nine genes, one Ran gene has been described previously [32]. The cDNA of *NlRSGs* contains an entire ORF range from 540 to 845bp in length. The predicted molecular weights of these proteins range from 20.2 to 30.3 kDa, with PI from 4.78 to 9.51. Sequences cloned in the present study were submitted to Genebank and their accession numbers, length of ORF, number of exon, gene loci, the PI and molecular weight (MW) of deduced amino were listed in Table 1. Based on sequence homology and domain organization, NlRSGs were divided into five subfamilies: Rab subfamily (including 14 genes), Rho subfamily (including 1 Rho, 1 Rac and 1Cdc42), Ras subfamily (including 1 NlRheb and 1 K-Ras), Ran subfamily and Arf subfamily (including 1 Sar1, 1 SRβ, 3 Arf and 4Arl).

**Table 1:** Ras-family genes in *N*.*lugens* and their sequence characteristics.

Gene name	Accession Number cDNA	ORF (bp)	exon NO.	Length of Gene	Gene loci (ORF coordinates)	MW (D)	PI
NlRab30	KT984790	612	5	12365	KN152431.1(1–612)	23.3	5.63
NlRab35	KT984791	594	5	13318	KN152285.1(1–594)	22.5	8.54
NlRab32	KT984792	723	5	12678	KN153722.1(1–740)	27.5	8.57
NlSar1	KT984793	585	5	12895	KN152447.1(1–585)	21.8	6.5
NlRab18	KT984794	624	5	8067	KN151947.1(1–624)	23.5	5.51
NlRab8	KT984795	627	5	20300	KN154377.1(1–627)	23.9	9.05
NlRab2	KT984796	639	5	11732	KN152218.1 (1–639)	23.7	6.35
NlRab7	KT984797	621	3	9906	KN153079.1(1–621)	23.3	5.3
NlRab11	KT984798	574	5	11871	KN152751.1(1–574)	24.4	5.8
NlK-Ras	KT984799	567	7	23167	KN153363.1(1–567)	21.5	7.3
NlRho	KT984800	582	/	/	KN152640.1(36–572)	21.8	8.03
NlRab23	KT984801	705	/	/	KN152203.1(33–705) KN155805.1(33–517) KN152218.1(1–32)	26.5	6.6
NlRab1	KT984802	615	/	/	/	22.8	4.78
NlRab14	KT984803	648	6	6184	KN152075.1(1–648)	24.2	6.74
NlArf1	KT984804	549	3	6884	KN152117.1(1–549)	20.7	6.4
NlRab7L	KT984805	845	5	7002	KN152606.1(1–845)	30.3	5.82
NlCdc42	KT984806	576	5	9632	KN152251.1(1–576)	21.2	6.38
NlArf6	KT984807	603	4	11432	KN152265.1(1–603)	22.8	9.51
NlArl3	KT984808	546	/	/	KN152926.1(1–443) KN152926.1(467–546)	20.4	8.8
NlArf2	KT984809	540	1	540	KN152837.1(1–540)	20.4	8.39
NlRheb	KT984810	549	5	21602	KN152467.1(1–549)	20.5	5.66
NlArl5B	KT984811	540	/	/	KN154202.1(1–255) KN153648.1(254–540)	20.4	6.01
NlRab6	KT984812	627	/	/	/	23.5	4.95
NlArl2	KT984813	555	5	5377	KN154257.1(1–555)	20.9	8.57
NlRab39	KT984814	660	4	11601	KN152962.1(1–660)	24.9	5.63
NlArl1	KT984815	543	3	5275	KN153275.1(1–543)	20.2	5.96
NlRac	KT984816	579	6	15368	KN153087.1(1–579)	21.5	7.92
NlSRβ	KU568374	762	5	15195	KN151966.1(1–762)	28.3	8.97
NlRan	KT313028	642	4	4147	KN153157.1(1–642)	24.5	7.31

bp,base pair;MW,Molecular weight;D,Dalton. Gene loci are obtained from blasting on database *N*. *lugens* genome (GenBank assembly GCA_000757685). The high identity region of ORF with genome was given in bracket.

Analysis of the genomic position and structure showed that the twenty-six *NlRSGs* ORF sequences were aligned to *N*.*lugens* genome, with *NlRab1* and *NlRab6* remained on as yet unmapped scaffolds. Interestedly the deduced amino acid of the two genes has the lowest PI with 4.78 and 4.95 respectively. The sequence from 1 to 255 of *NlArl5B* was matched on KN154202.1 (full length 139047bp) of *N*.*lugens* genome between 109344 to 100169, while from 254–540 was matched on KN153648.1(full length 205899bp) between 29534 to 31028. So we proposed that KN154202.1 and KN153648.1 of *N*.*lugens* genome are in same scaffold. The ORF from 1–35 of *NlRho* showed no similarities in *N*.*lugens* genome. Because of the gap of genome in *N*.*lugens*, the number of copy or exon in *NlRab1*, *NlRab6*, *NlArl5B*, *NlRho* and *NlRab23*, *NlArl3* was uncertain. The gene length and loci corresponding on *N*. *lugens* genome were listed in Table 1.

**Fig 1:**
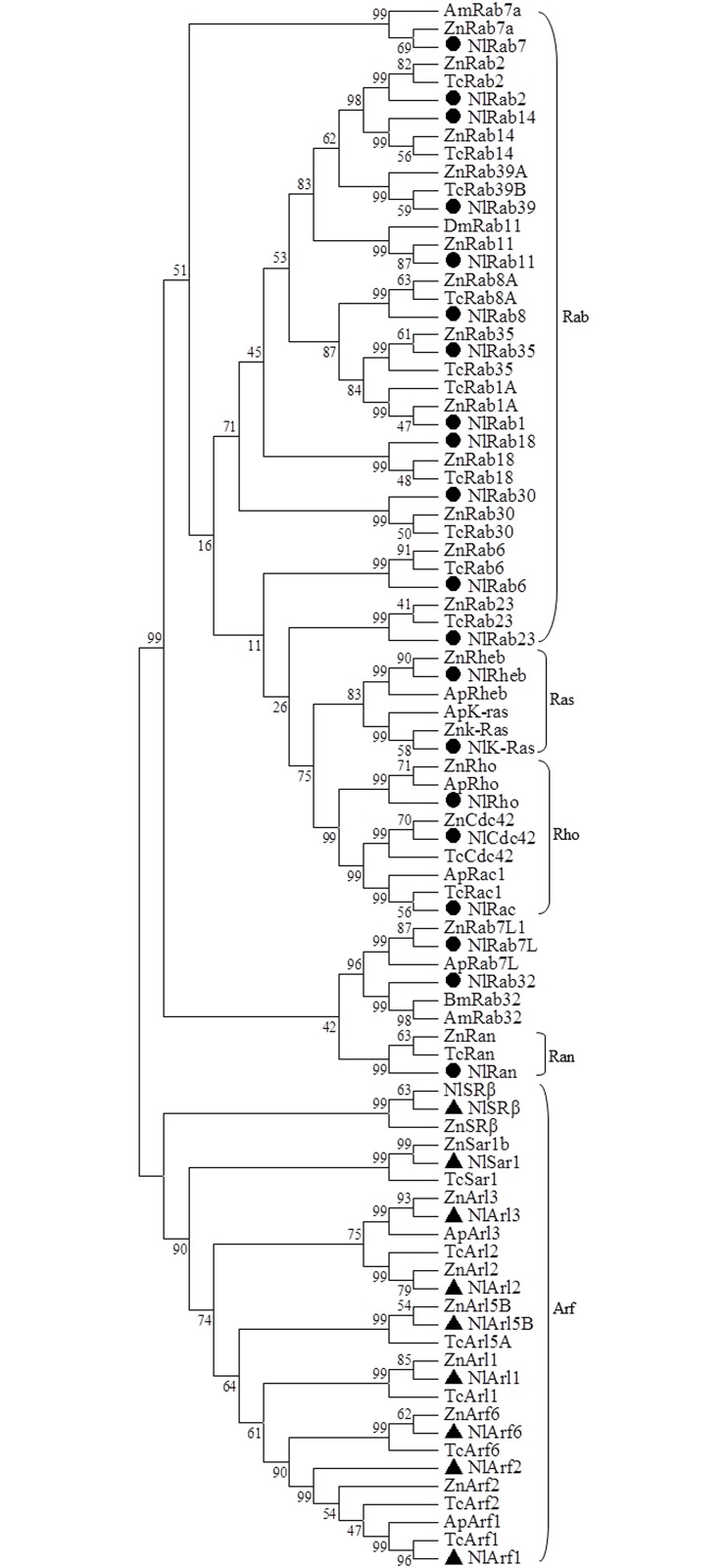
Unrooted phylogenetic tree of RSGs from *N*.*lugens* and representative insect species. An unrooted phylogenetic tree was constructed by the neighbour-joining method, together with 1000 bootstrap replicates based on the protein sequence alignments. *N*.*lugens* proteins in Class I were marked black circle. *N*.*lugens* in Class II was marked with black triangle. Gross Ras subfamily groupings were shown on the right. (See S2 Table for details of GeneDB accession numbers).

Phylogenetic tree was constructed using the NJ method to evaluate the molecular evolution relationships of the twenty-nine RSGs proteins of *N*.*lugens* and other RSGs from representative insect species. In the phylogenetic tree, twenty-nine NlRSGs were clustered into two classes. Rab, Ran, Rho and Ras were clustered together, Rab32 and Rab7L fall slightly outside the main Rab cluster. Sar1, SRβ and Arf formed another separate class. SRβ is distantly related to Arf and Sar1. Rab proteins represent the largest branch of the RSGs gene (Fig 1). Except for the genes *NlRab1*, *NlRab6*, *NlArl5B*, *NlRho*, *NlRab23* and *NlArl3* that are absent or do not have enough genomic information, the structure of the other twenty-three genes was characterized and shown to contain between 0 and 6 introns (S1 Fig). Among twenty-three RSGs genes, only *NlArf2* possesses no intron, which is similar with that from *A*.*pisum*, *Tribolium castaneum* and *A*.*mellifera*. Other *NlRSGs* genes were divided into many segments by introns. Introns are located either between codons (phase 0) or within codons (phase 1 and 2) [33].

**Fig 2:**
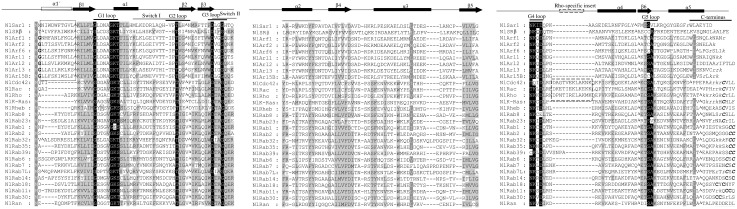
Amino acid sequence alignment of *N*.*lugens* RSGs proteins. ClustalX (2.1) program was used for multiple sequence alignment. G loop consensus residues were highlighted in black. C-terminal region cysteines were highlighted in bold italic and C-terminal basic residues (K and R) were highlighted in lowercase italic. The highly acidic C-terminal motif DEDDDL in NlRan was underlined. N-terminal glycines in positions favoring myristoylation were highlighted in bold. Gray highlighting indicated residues that were highly conserved in 90% of members. G loop and C-terminal were highlighted in black line. Switch regions were highlighted in gray line. The highly conserved residue arginine and proline, an important hallmark of Rheb and SRβ respectively, distinguishing them from other small GTPases were highlighted in frame. Rho-specific insert and α1′helix in Arf subfamily were framed with dashed lines. Secondary-structure elements were marked with arrows (β strands) and filled rectangles (helices). Amino acid omitted for optimum alignment was indicated with the ۸ symbol. Gaps have been introduced to permit alignment.

Deduced amino acid sequence showed more than 76% identity with their respective amino acid of other insects except NlSRβ and NlRab7L. The best matched sequence for NlSRβ is that SRβ from *Megachile rotundata*, with 56% identity and 90% coverage at amino acid level, 68% identity and 56% coverage at nucleotide level. The best matched sequence for NlRab7L is that Rab7L from *Halyomorpha halys*, with 67% identity and 82% coverage at protein level, 75% identity and 62% coverage at nucleotide level. The multiple alignments of twenty-nine *N*.*lugens* proteins were shown in Fig 2. All these newly cloned proteins have four conserved GTP-binding or GTPase regions of the small G protein superfamily (G1, G3, G4, and G5 loop), as well as an effector site (G2 loop) and a universal conformational switch (switch I and II) [11]. RSGs consist of six-stranded β sheet (β1–β6), five parallel strands (α1–α5) according to the three-dimensional structures [34, 35]. Transmembrane helix was predicted only in protein NlSRβ at residues 21–43 as reported by Miller [36]. Rho family members (Rho, Rac and Cdc42) are defined by the presence of a Rho-specific insert about 13 amino acids located between the G4 and the G5 loop and involved in the binding to effectors and regulators [37]. Another important biochemical feature of a majority of RSGs is their post-translational modification by lipids. The modification dictates their specific subcellular locations and interactions with distinct membrane compartments. The C-termini motif of Rab, Ras and Rho and N-termini glycines of Arf family are modified by lipids. The C-termini motif of RSGs from *N*.*lugens* differ in length from 27 to 43 and can be divided into five types Cxxx, CC, CxC, CCx or CCxxx, where x is any other amino acid(Fig 2).

### The expression of *NlRSGs* genes in *N*.*lugens*

The expression of all *NlRSGs* did not show obvious variation at various developmental stages. The highest expression level was found in newly emerged adults and majority of the *NlRSGs* genes showed higher expression level in females than in males (data not shown).

**Fig 3:**
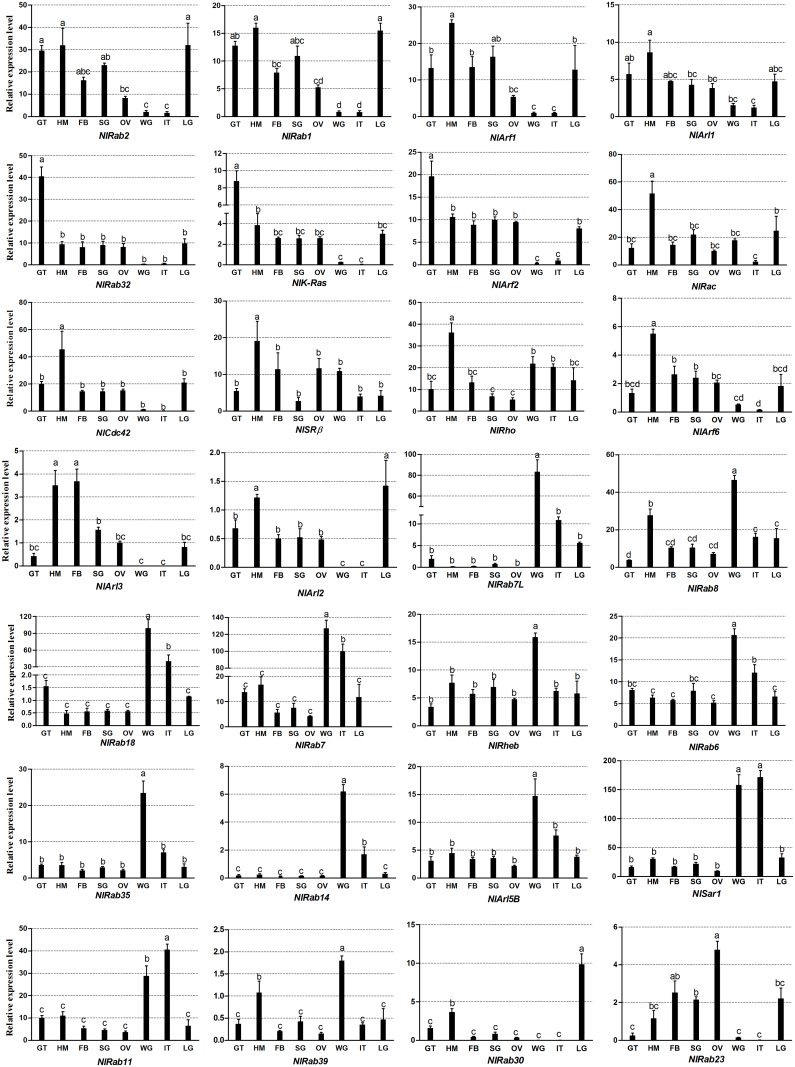
Tissue expression patterns of *N*. *lugens* genes encoding RSGs. Total RNA was extracted from the salivary gland (SG), gut (GT), fat body(FB), ovary(OV), hemolymph(HM), integument(IT), leg(LG) and wing (WG). Three biological replications (mean ± SE) were carried out for each gene based on independent samples and relative value for the expression level of target gene was calculated by the equation Y = 10 ^(Ct internal- Ct target)/3^ x100%, using the geometric mean of 18S rRNA and β-actin as internal control. Different letters indicate significant differences among different tissues by Duncan’s multiple range test at P < 0.01.

The results of qRT-PCR in tissue showed that the *NlRSGs* expressed in all tested tissues, including gut, haemolymph, fat body, salivary gland, ovary, integument, leg and wing, except *NlArl2* was not expressed in integument and wing. In addition, three *NlRSGs* (*NlRab32*, *NlK-Ras* and *NlArf2*) expressed with the highest level in gut. Five *NlRSGs* expressed with the highest level only in haemolymph. Eleven *NlRSGs* expressed with the highest level in wing. A particularly high expression level of *NlArl2* was found in haemolymph and leg. *NlArl3* showed higher expression level in fat body and haemolymph than that in other tissues. *NlRab23* expressed highly in ovaries (Fig 3). The different expression pattern in tissues may provide clues of the biological functions of *NlRSGs* in *N*.*lugens*.

### Expression of target genes is suppressed by the injection of its specific dsRNA

**Fig 4:**
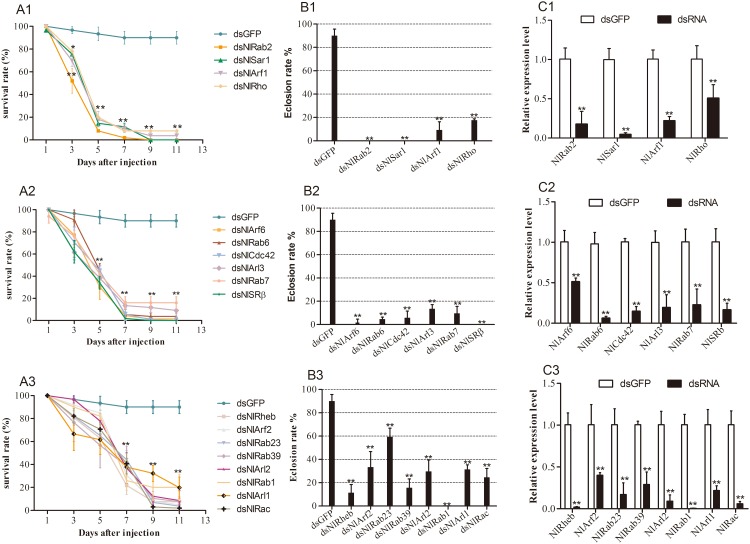
Injection of dsRNA on survival (A1-A3), eclosion (B1-B3) of *N*. *lugens* and mRNA level of target gene (C1-C3). The third instar nymphs were injected with specific dsRNA and were observed for phenotypic variations at 48h intervals. Individuals treated with dsGFP were used as a control. The survival and eclosion rate were calculated from three biological replicates (mean ± SD). For each treatment, n = 30 nymphs. mRNA level of target gene at 4d after injection in *N*.*lugens* were analyzed by qRT-PCR with 2^-ΔΔCt^ method from three biological replicates (n = 5, mean ± SD). **P < 0.01, * P < 0.05.

About 454-898bp dsRNA fragments were injected into 3^th^ instar nymph respectively to ascertain the biological function in insect development. There was no difference at the gene expression level between nymphs injected with double distilled water and dsGFP, so we use the nymphs injected dsGFP as control. After dsRNA injection, mRNA abundance of target gene in the survival nymphs was examined by qRT-PCR. The mRNA level of *NlCdc42* and *NlSRβ* was investigated over seven days after the injection. As expected, *NlCdc42* expression levels in nymphs decreased by 68.7%, 75.9%, 65.9%, 91.6%, 84.0%, 83.5% and 82.5% respectively (S2 Fig). *NlSRβ* expression levels in nymphs decreased by 4.3%, 72.8%, 68.6%, 83.8%, 77.2%, 80.7% and 80.1% respectively, when compared with that in dsGFP- injected nymphs (S2 Fig). The relative expression of *NlSRβ* expression was not reduced in one day after dsRNA injection. The maximum reduction for target gene was occurred at the 4^th^ day after injection. Because of high mortality, there were no enough samples for analysis the RNAi efficiency from the seventh day onward. The RNAi efficiency was demonstrated for other *NlRSGs* genes at the 4^th^ day post injection. At the same concentration (70ng/nymph) of injected dsRNA, qRT-PCR showed that the transcript level of target genes reduced by 39.2%-99.5%, comparing with the dsGFP control. Among the eighteen *NlRSGs* which have lethal effect in *N*.*lugens* when silenced, the expression level decreased more than 60.0% for fifteen *NIRSGs* genes (Fig 4C1–4C3). Less than 50% decrease for gene *NlRho* (Fig 4C1), *NlArf6* (Fig 4C2) and *NlArf2* (Fig 4C3).

### RNAi screen to identify NlRSGs genes with crucial functions in the development of *N*.*lugens*

**Fig 5:**
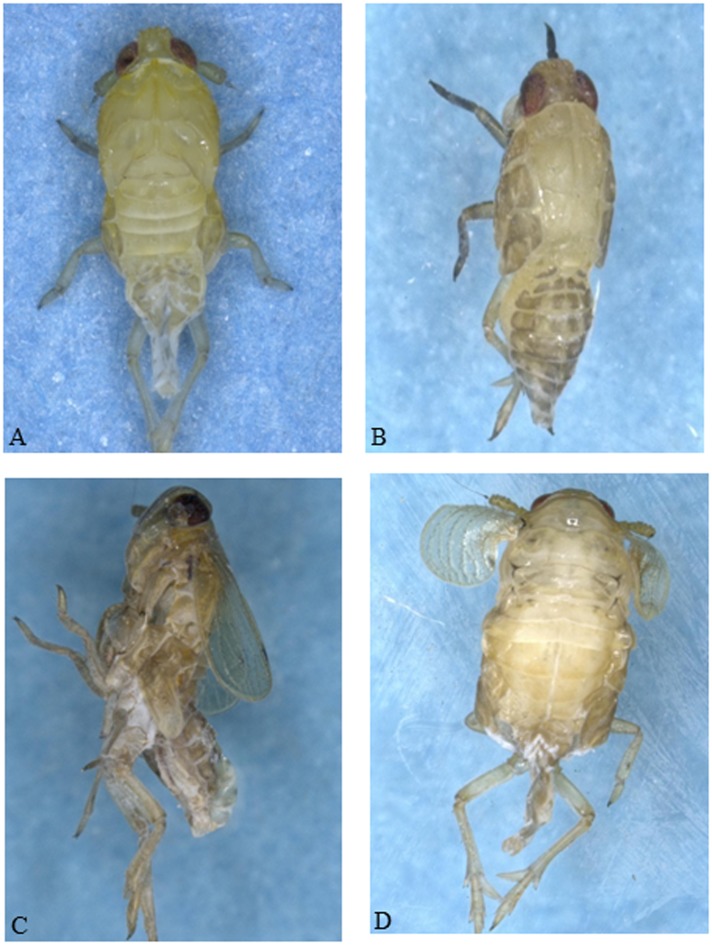
Effect of dsRNA injection on the nymphal molting. The typical phenotype of nymphs with silenced *NlSRβ*, *NlSar1*, *NlArf6* or *NlRab2* (A, B) which did not molt into next stage and old nymphal exoskeletons remained on the tips of legs and abdomens and died later. The typical phenotype of nymphs with silenced *NlRheb*, *NlRab1*, *NlRab39* or *NlRac* (C, D) which started to ecdyse but failed to shed the exuvia completely and showed deficiencies in the extension of the wings.

To examine the role of individual *NlRSGs* in the nymphal development of *N*.*lugens*, we synthesized dsRNA for twenty-eight *NlRSGs* genes and lowered its expression by RNAi individually. The phenotypic defects in morphological and lethal characteristics were almost not detectable in dsGFP-injected or water-injected nymphs throughout the test period. The nymphs in the water or dsGFP controls were successfully molted to adult at 5^th^ day and 90.0% individuals were survival to adult at 11^th^ day after injection. In contrast, a large-scale RSGs RNAi screen identified the suppression of eighteen *NlRSGs* transcription levels resulted in lethal effect in *N*.*lugens*. The survival rate of nymphs began to decrease 3 days after injecting dsRNA of *NlRab2*, *NlSar1*, *NlArf1* or *NlRho*. The surviving rate was 52.0–78.0% at 3^rd^ day, which dramatically dropped to less than 20.0% at 5^th^ day, Nine days after injecting dsRNA of *NlRab2* or *NlSar1*, the survival rate of nymphs decreased to 0.0%, yet the dsGFP control remained high (90.0%)(Fig 4A1). The survival rate of nymphs began to decrease 3 days after injecting dsRNA of *NlRab6*, *NlCdc42*, *NlSRβ*, *NlRab7*, *NlArf6* or *NlArl3*. The surviving rate was 30.0–45.0% at 5^th^ day, which dramatically dropped to less than 15% at 7^th^ day after injection. Nine days after injecting dsRNA of *NlSRβ*, the survival rate of nymphs decreased to 0.0% (Fig 4A2). More than 80.0% of dsNlArf2 and dsNlRab1-treated nymphs survived at 5^th^ day after injection. However, the surviving rates decreased at 7^th^ day and dramatically declined to less than 10% and 20% respectively at 11^th^ day after injection (Fig 4A3). No nymphs with injected dsNlSRβ, dsNlRab2, dsNlSar1 or dsNlRab1 eclosed to adult. Less than 17.6% of dsNlRab7, dsNlRab6, dsNlRab39, dsNlRheb, dsNlRho, dsNlCdc42, dsNlArf1, dsNlArf6 or dsNlArl-treated nymphs were survived to ecose (Fig 4B1 and 4B2). Many of the deaths occurred during molting stage in nymphs with silenced *NlRab2*, *NlSar1*, *NlArf6* or *NlSRβ*. A typical phenotype is that the nymphal cuticles remained on the tips of legs and were not completely shed (Fig 5A and 5B). Many of the deaths occurred during nymph-adult ecdysis in nymphs with silenced *NlRheb*, *NlRab39*, *NlRab1* or *NlRac*. The typical phenotype was that the wings wrinkled, puckered or bent and the nymphal cuticles cannot be completely drained away (Fig 5C and 5D). About 59.3% nymphs with silenced *NlRab23* survived to adult emergence, the mortality at 11^th^ day was high to 96.3% (Fig 4A3). This suggested that *NlRab23* plays an important role in nymph and adult development. About 20% nymphs with silenced *NlRab1* were survival at 11^th^ day, while no nymphs can adult eclosion (Fig 4A3 and 4B3). So we proposed *NlRab1* plays an important role in nymph-adult transition. By contrast, the suppression of other ten genes did not lead to abnormal effects in *N*.*lugens*, although more than 90% decrease in the transcript levels following RNAi application were observed.

### Function of *NlRSGs* in the 20E and JH signal pathway

**Fig 6:**
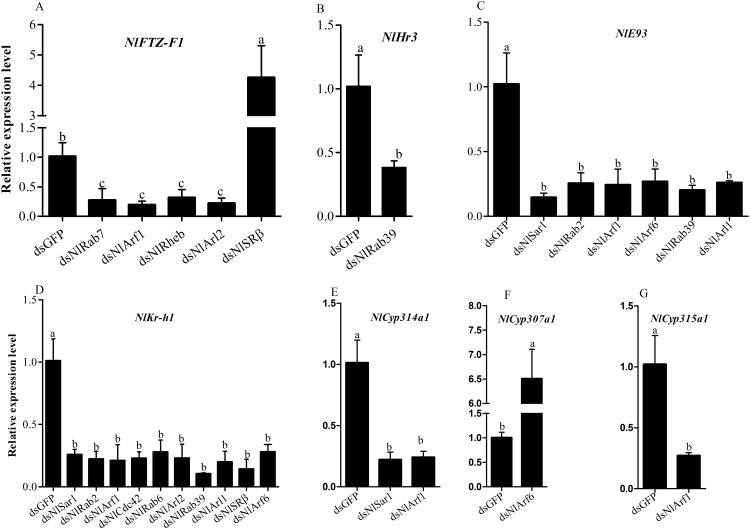
qRT-PCR analysis of genes expressions in the nymphs injected with dsGFP and dsRSGs. Error bars represent the standard deviation in three independent RNAi experiments. Different letters indicate a significant difference at P <0.05.

JH and 20E play critical signaling roles during insect development and adulthood and are essential for normal metamorphosis and reproduction [38, 39]. To examine the possible function of RSGs in the 20E or JH signal pathway, we conducted qRT-PCR experiment to examine expression levels of eight nuclear receptor genes involved in the 20E and JH signal pathway and five Halloween genes involved in 20E biosynthesis in nymphs with silenced *NlRSGs*. Of those eighteen genes which have lethal effect on nymph development after dsRNA injection, twelve genes were proposed to be related with 20E or JH singal pathway. The expression of *NlFTZ-F1*, *NlHr3*, *NlKr-h1* or *NlE93* and three Halloween genes were significantly decreased or increased when compared to their mRNA levels in the nymphs with dsGFP (Fig 6A–6G). The expression level of *NlKr-h1* was decreased by 89.6–74.4% in nymphs with silenced eight genes respectively (including *NlSar1*, *NlRab2*, *NlArf1*, *NlCdc42*, *NlRab6*, *NlRab39*, *NlArl1* or *NlSRβ*). The expression level of *NlE93* was decreased by 85.6–74.6% in nymphs with silenced six genes respectively (including *NlSar1*, *NlRab2*, *NlArf1*, *NlArf6*, *NlRab39 or NlArl1*) as compared to nymphs injected with dsGFP. From our result, we conclude that the expression of at least two nuclear receptors was decreased in nymphs with silenced *NlRab2*, *NlArl1* or *NlArl2*. At least the expression of three genes including nuclear receptors and Halloween genes was decreased in nymphs with silenced *NlSar1* and *NlRab39*. The nymphs with silenced *NlArf1* demonstrated 4–5 folds reduction in five genes expression including two Halloween genes *NlCyp315a1* and *NlCyp314a1* and three nuclear receptors *NlKr-h1*, *NlFTZ-F1* and *NlE93*. The nymphs with silenced *NlSRβ* demonstrated an approximate 3.3 folds reduction in *NlKr-h1* gene expression. In contrast, the gene expression of *NlFTZ-F1* was increased about 5 folds when compared with the controls. The nymphs with silenced *NlArf6* demonstrated an approximate 3–4 folds reduction in *NlKr-h1* and *NlE93*, while the transcript level of *NlCyp307a1* was increased about 6.5 folds. The transcript level of other nuclear receptors including *NlECR*, *NlMet*, *NlBr-C* and *NlE75* and Halloween genes *NlCyp306a1*, *NlCyp302a1* was only slightly or without influenced in RNAi-treated nymphs (data not shown). Of those eighteen genes, down-regulation of *NlRho*, *NlArl3*, *NlRab1*, *NlArf2*, *NlRab23* and *NlRac* has no significant effect on transcript level of tested nuclear receptors and Halloween genes.

### Identification of efficient RNAi target genes for *N*.*lugens* control

Three potential effective RNAi target genes *NlRab2*, *NlSar1* and *NlSRβ* genes, which were marked by 0.0% eclosion rate and almost 100.0% mortality on day nine post injection, were extensively studied by alignment protein sequences from seven insects including *A*.*pisum*, *T*.*castaneum*, *A*.*aegypti*, *Z*.*nevadensis D*.*melanogaster*, *B*.*mori* and *A*.*mellifera*. The results indicated that NlRab2 andNlSar1 are both highly conserved among different insects (S3 and S4 Figs). However, NlSRβ is not highly conserved among different insects (S5 Fig). The analysis of sequence pair distances with Megalign software suggested that NlSRβ share relatively low sequence similarity with other insects, while sequence identity at both the nucleotide and the amino acid level is very high for NlSar1 and NlRab2 (Table 2). And then we searched the nucleotide sequences against the well annotated NCBI transcriptome databases for >19 bp long stretches of identity. The result showed no ≥19nt overlap (data not shown), and thereby prevented RNAi-induced silencing in the non-target species.

**Table 2:** Sequence pair distances of three genes.

Gene name	Dm	Am	Bm	Aa	Zn	Tc	Ap
*NlRab2*	89.2/75.6	88.7/64.0	88.2/72.8	90.1/73.1	95.8/77.9	91.0/72.0	89.6/70.6
*NlSar1*	63.2/62.1	69.6/65.3	73.5/70.3	73.7/70.1	68.8/66.0	71.3/65.8	70.1/63.0
*NlSRβ*	29.4/37.3	53.2/47.2	38.8/39.8	37.2/34.6	51.3/44.2	45.1/43.1	47.9/41.3

Sequence pair distances were calculated by the ClustalV method of Megalign software between insects. Degree of identity at protein level in percentage is given in left of slash and the degree of identity at DNA level in percentage is given in right of slash. The data sources for all Ras family GTPase are listed in S3 Table. Ap, *Acyrthosiphon pisum*;Bm, *Bombyx mori*;Zn, *Zootermopsis nevadensis*;Aa, *Aedes aegypti*;Dm, *Drosophila melanogaster*;Am, *Apis mellifera*;Tc, *Tribolium castaneum*.

**Fig 7:**
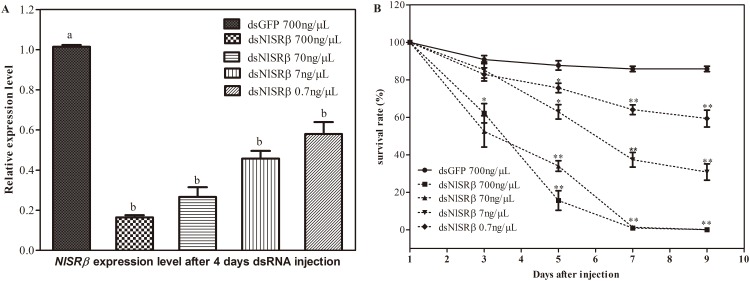
NlSRβ mRNA abundance (A) and survival (B) of *N*. *lugens* that treated with different concentrations of dsNlSRβ. mRNA level of *NlSRβ* at 4d after injection were analyzed by qRT-PCR with 2^−ΔΔCT^ method from three biological replicates (mean±SD, N = 5). Different letters indicate a significant difference at P <0.05. The survival rate was calculated from three biological replicates (mean±SD, N = 30 nymphs). *P < 0.05, **P < 0.01 by Duncan’s multiple range test.

To determine the sensitivity of *N*.*lugens* to dsNlSRβ, different concentration gradient experiments were performed. In the third-instar nymphs, injection of dsNlSRβ (0.1μL) at the concentrations of 0.7, 7.0, 70.0 and 700.0ng/μL suppressed *NlSRβ* expression level by 42.9%, 55.0%, 73.7% and 83.7%, respectively, comparing with the levels in dsGFP injected nymphs(Fig 7A). The survival rates decreased from 3^rd^ day and dramatically dropped to 0.93% and 1.3% at the 5^th^ day for nymphs injected with concentrations of 70.0 and 700.0ng/μL dsNlSRβ, respectively. No nymphs were survived at the 9^th^ day. As the dose of dsNlSRβ used decreased to 7.0 and 0.7 ng/μL, the survival was increased and reached to 30.7% and 59.4% at the 9^th^ day respectively (Fig 7B). The result suggested that no nymphs were survived in the dsNlSRβ-treated group when the dose of dsNlSRβ given was higher than 7ng/per insect.

## Discussion

The RSGs take part in numerous and diverse cellular processes and act as switches to regulate protein activity or localization. Protein localization to their correct subcellular compartments is critical for eukaryotic cells to maintain the high degree of order required for life.

### Identification of RSGs genes in *N*. *lugens*

In yeast, *Trypanosoma brucei*, *C*.*elegans*, *D*.*melanogaster* and humans, the number of genes for RSGs is 30, 40, 56, 90, and 174, respectively [40, 41]. In this study, we cloned the cDNA of twenty-nine RSGs from *N*.*lugens*. These proteins were clustered into two class and five subfamilies. The largest class is the Rab family with 14 members followed by Arf, with 11 members including Sar1 and SRβ. Homology analysis revealed that NlRSGs were conserved among different insect species. This suggests that the biological functions may be similar to the corresponding RSGs in other model organisms. Sequence alignments showed that all NlRSGs shared a common G domain, two switch regions (I and II) and significant sequence similarity. The five types cysteine motif boxes (Cxxx, CC, CxC, CCx or CCxxx) and C-termini polybasic region of Rab, Ras and Rho play important roles in subcellular localization and small GTPase function[42,43]. The Arf family proteins are characterised by being anchored to the bilayer in their GTP-bound state by an N-terminal myristoylation at Gly2 found at the N-terminal amphipathic helix (α1′helix) rather than the C-terminal lipid anchor used by most other members of the Ras superfamily of small G proteins [44–46]. These acylations localize the RSGs to cellular membranes, where their signaling activities take place [43]. Sar1 lacks N-myristoylation but has a highly conserved bulky hydrophobic patch (NIW in NlSar1) within N-terminal amphipathic helix that mediates recruitment of Sar1 to ER membranes and can also induce their curvature in vitro [47, 18]. In contrast to Arf proteins, SRβ is a membrane-bound protein via its N-terminal transmembrane helix and has a distinct sequence-conservation pattern in the switch II region. The proline residues in switch II region is phylogenetically well conserved in all SRβ orthologues, which undergoes cis/trans isomerization as part of this G protein switch [48]. Transmembrane helix was predicted in protein NlSRβ at residues 21–43. The proline residues of 102 position in NlSRβ is strictly conserved as glycine in the Arf-type proteins. This subtle difference to the Arf-type switch suffices to create a quite different switch mode that regulates the protein targeting process. Rans are closest to Arfs. They also lack cysteines at the C-ends and are not posttranslationally modified at all. Ran has an elongated COOH-terminal element (EDDEDL (209–214) in NlRan) crucial for its function in nuclear transport [35].

Tissue specificity analysis showed the various expression patterns of RSGs genes in *N*.*lugens*. Three *NlRSGs* expressed at the highest level in gut. Five *NlRSGs* expressed at the highest level only in haemolymph. Since gut is an important organ related to the absorption and transport of nutrients. Intracellular transport is more active here than in other tissues. Haemolymph plays a central role in insect immune response and defend against pathogen invasion. RSGs are postulated to be implicated in the hemocyte cellular processes to perform phagocytosis, nodulation, and encapsulation behaviors [19, 49]. This might indicate that the possible intracellular transport or immune-related functions of *NlRSGs* which highly expressed in the gut and haemolymph. The wing we dissected included the direct flight muscles at the base of the wing. Our result showed that eleven *NlRSGs* (eight belong to Rab subfamily) expressed with the highest level in wing. Insect wing muscle is a strictly aerobic tissue and contains a large number of endoplasmic reticulum or sarcoplasmic reticulum [50]. Rab GTPases associate with distinct endomembrane compartments and are essential for the transport of proteins and membrane through the endomembrane system to their destination [51]. The *NlRSGs* highly expressed in wing may be associated with ER and involved in functional regulation of muscles and wings.

### RNAi induces target gene repression and lethal effect in *N*. *lugens* nymphs

When nymphs were injected with dsRNA, levels of transcripts of the targeted genes were reduced. RNAi revealed the functional importance of eighteen *NlRSGs* in nymphal development, which showed lethal effect. Most nymphal death was occurred during nymph molting or nymph-adult ecdysis. While the other ten genes were not the key factor in nymphal development of *N*.*lugens*. RNAi studies showed that some Rab subfamily genes such as *NlRab2*, *NlRab7*, *NlRab6 NlRab23*, *NlRab1* and *NlRab39* are essential, whereas others are dispensable. They may have only partially overlapping functions in membrane traffic. Although only 39.2–49.5% decrease of gene expression was observed for RNAi of *NlArf6*, *NlArf1* and *NlRho*, the adverse effect on *N*.*lugens* implies the importance of the three genes in *N*.*lugens* development. All the Arf subfamily genes except *Arfl5B* and Rho subfamily genes are essential for *N*.*lugens* development. We demonstrated that NlRab2, NlSar1 or NlSRβ is an indispensable factor during the molting or eclosion process. The silencing of *NlRab2*, *NlSar1* or *NlSRβ* expression which highly expressed in tissue haemolymph, leg, wing or integument leads to nymphal cuticles shed incompletely and 100% mortality at 9^th^ day.

In insects, the life cycle is characterized by a series of moltings, including larval/nymph molting and metamorphic molting. The molting process is regulated by steroid hormones including 20E and JH acting via nuclear receptors [39]. Five halloween genes coding for the P450 enzymes that control the 20E biosynthesis pathway. Nuclear receptors EcR, Br-C, E75, Hr3, FTZ-F1 and E93, Kr-h1 participated in 20E and JH regulatory cascade respectively [52]. And also the appropriate timing of their expression is almost certainly essential for their correct function [52]. Knock-down of some *NlRSGs* inhibited nymphs molting or nymphal–adult transition. The phenotypic defects similar to insect whose ecdysteroid-mediated signaling had been inhibited [53–56] or whose ecdysteroid synthesis had been disturbed [57, 58]. Therefore the transcription level of nuclear receptors and halloween genes were tested in RNAi-treated nymphs. RNAi of twelve *NlRSGs* individually resulted in the transcriptional down or up-regulation of nuclear receptors (*Kr-H1*, *Hr3*, *FTZ-F1* and *E93)* or ecdysteroid synthesis genes (*Cyp307a1*, *Cyp314a1* or *Cyp315a1*) in *N*.*lugens*. When the gene *NlArf1* was suppressed, not only the expression of *NlArf1* decreased, but also the expressions of other genes involved in the 20E biosynthesis and signal transduction pathway were affected, such as *Cyp315A1*, *Cyp314a1*, *E93*, *Kr-h1* and *FTZ-F1*. When the gene *NlSar1* was suppressed, the expression of *Cyp314a1*, *E93* was affected. While the gene *NlArf6* was suppressed, the expression of *Cyp307a1*, *E93 and Kr-h1* was affected. These results suggested that *NlArf1*, *NlArf6* and *NlSar1* are involved in regulation of transcription of 20E biosynthesis genes and nuclear receptors. While most *NlRSGs* are necessary for nuclear receptors involved in the transcription of 20E or JH signal pathway. Different members of the RSGs can activate multiple signaling pathways leading to the activation of transcription factors, such as the activation of nuclear factor by members of Rho family [59, 60]. We proposed that the problems to complete the molting process in dsNlRSGs insect were partially due to disruption of the steroid hormones signal pathway via a) failure regulatory function on ecdysteroid synthesis or nuclear receptors transcription; b) destruction the consistence and appropriate timing expression patterns; c) improper localization proteins participated in ecdysteroid synthesis or signal pathway.

### *NlSRβ* a better candidate as a target for pest control purpose

To discover potential RSGs as pesticide targets, a large-scale RNAi screen was performed by injecting dsRNA into developing nymph in this study. Eighteen RSGs were found involved in nymphal growth, molting and metamorphosis. The identified RSGs may serve as potential insecticide targets for controlling *N*.*lugens* and other related pest species. Among these eighteen RSGs genes, severe RNAi-induced phenotypes were observed for *NlSRβ*, *NlSar1* or *NlRab2*. Specifically, knock-down resulted in 100% mortality and 0% eclosion rate within 9 days. In order to protect non-target organisms it would be desirable to use dsRNA fragments that are specific to the pest species and do not contain sequences targeting genes in non-target organisms (off targets). dsRNA are processed by the enzyme Dicer into 21-23nt long short interfering RNAs, they serve as template to recognize the complementary mRNA and target it for destruction [61]. This short interfering RNAs with an exact sequence identity of ≥19nt can already induce off target effect [62]. Therefore, the sequence conservation of three potential RNAi target genes were analyzed. The analysis suggested *NlSRβ* could be served as a target for dsRNA-based pesticides for *N*.*lugens* control. Firstly, *NlSRβ*, as an integral membrane protein, is constitutively expressed in almost all tissues. The SRβ is required for the cotranslational targeting of both secretory and membrane proteins to the ER membrane [36]. Protein translocation across and insertion into membrane is essential to all life forms. It is not surprising, therefore, that any changes in SRβ expression and function have profound effects on many cellular functions. Secondly, injection only 7.0 ng/nymph dsRNA of *NlSRβ* during nymphal development was sufficient to induce 100% mortality and hindered eclosion completely. The expression of Kr-H1 and FTZ-F1 was interefered by RNAi of *NlSRβ*. Thirdly, NlSRβ shared relatively low sequence similarity (34.6–44.2%) over the entire gene’s length and showed no >19 bp overlap with other insects, thereby it was relatively easy to design dsRNA sequences that were unique to *N*.*lugens* and prevented RNAi-induced silencing in the non-target species. Therefore, *NlSRβ* seems a better candidate as a target for pest control purpose.

In summary, we identified twenty-nine *NlRSGs* genes and demonstrated the importance of eighteen *NlRSGs* in nymphal development of rice pest *N*.*lugens*. *NlSRβ* was proposed to be a potential effective target for RNAi-based pest control. Attempts to analysis the function of *NlRSGs* in reproduction and cellular immune are in progress in our laboratory.

## Supporting information

S1 Fig Phylogenetic tree (A) and intron–exon structures (B) of RSGs genes in *N*.*lugens*. The number of branches on the phylogenetic tree indicated the bootstrap values. The phylogenetic tree was constructed by maximum likelihood based on the full-length cDNAs. cDNA and genomic sequences were compared putative exon–intron map. Exons and introns are indicated with the black boxes and black lines, respectively. The number indicated intron phase. Lengths are roughly at scale.
(TIF)

S2 Fig Silencing efficiency of injection dsNlCdc42 (A) and dsNlSRβds (B). The relative transcript level for each sample was measured daily from 3 independent pools of 5 nymphs. P < 0.01 was considered statistically significant (**) different from dsGFP treatment.
(TIF)

S3 Fig Amino acid sequence alignments of Rab2 from eight species. Blank residues indicate identical amino acids. Gray residues represent conserved substitutions. Sequences were aligned using ClustalW. Nl,*N*.*lugens*;Ap, *Acyrthosiphon pisum*;Bm, *Bombyx mori*;Zn, *Zootermopsis nevadensis*;Aa, *Aedes aegypti*;Dm, *Drosophila melanogaster*;Am, *Apis mellifera*;Tc, *Tribolium castaneum*. The data sources for all Ras family GTPase are listed in S3 Table.
(TIF)

S4 Fig Amino acid sequence alignments of Sar1 from eight species. Blank residues indicate identical amino acids. Gray residues represent conserved substitutions. Sequences were aligned using ClustalW. Nl,*N*.*lugens*;Ap, *Acyrthosiphon pisum*;Bm, *Bombyx mori*;Zn, *Zootermopsis nevadensis*;Aa, *Aedes aegypti*;Dm, *Drosophila melanogaster*;Am, *Apis mellifera*;Tc, *Tribolium castaneum*. The data sources for all Ras family GTPase are listed in S3 Table.
(TIF)

S5 Fig Amino acid sequence alignments of SRβfrom eight species. Blank residues indicate identical amino acids. Gray residues represent conserved substitutions. Sequences were aligned using ClustalW. Nl,*N*.*lugens*;Ap, *Acyrthosiphon pisum*;Bm, *Bombyx mori*;Zn, *Zootermopsis nevadensis*;Aa, *Aedes aegypti*;Dm, *Drosophila melanogaster*;Am, *Apis mellifera*;Tc, *Tribolium castaneum*. The data sources for all Ras family GTPase are listed in S3 Table.
(TIF)

S1 Table Primers used in this study. F,forward primer,R,reverse primer.
(DOC)

S2 Table Protein sequences sources used in Fig 1. (DOCX)

S3 Table Sequences sources used in Table 2 and S1 Fig. (DOC)

## Abbreviations

20E: 20-hydroxyecdysone
Arf: ADP-ribosylation factors
Arl: Arf-like proteins
dsRNA: double strand RNA
E93: Ecdysone-induced protein 93
FTZ-F1: Fushi tarazu factor-1
GDP: guanosine diphosphate
GTP: guanosine triphosphate
Hr3: Hormone receptor 3
JH: juvenile hormone
Kr-h1: Krüppel homolog 1
Met: Methoprene-tolerant
qRT-PCR: quantificational real-time polymerase chain reaction
Rac: Ras-related C3 botulinum toxin substrate
Ran: Ras-related nuclear proteins
Ras: Rat sarcoma
Rho: Ras homolog
RNAi: RNA interference
RSGs: Ras-like family small GTPases
Sar1: Secretion-associated and Ras-superfamily-related proteins
SRβ: beta subunit of the signal recognition particle receptor
