# Supplementary figures and images for: Ras-like family small GTPases genes in Nilaparvata lugens: Identification, phylogenetic analysis, gene expression and function in nymphal development
Source: PLoS One. 2017 Feb 27;12(2):e0172701. doi: 10.1371/journal.pone.0172701 (PMC5328259; doi:10.1371/journal.pone.0172701)

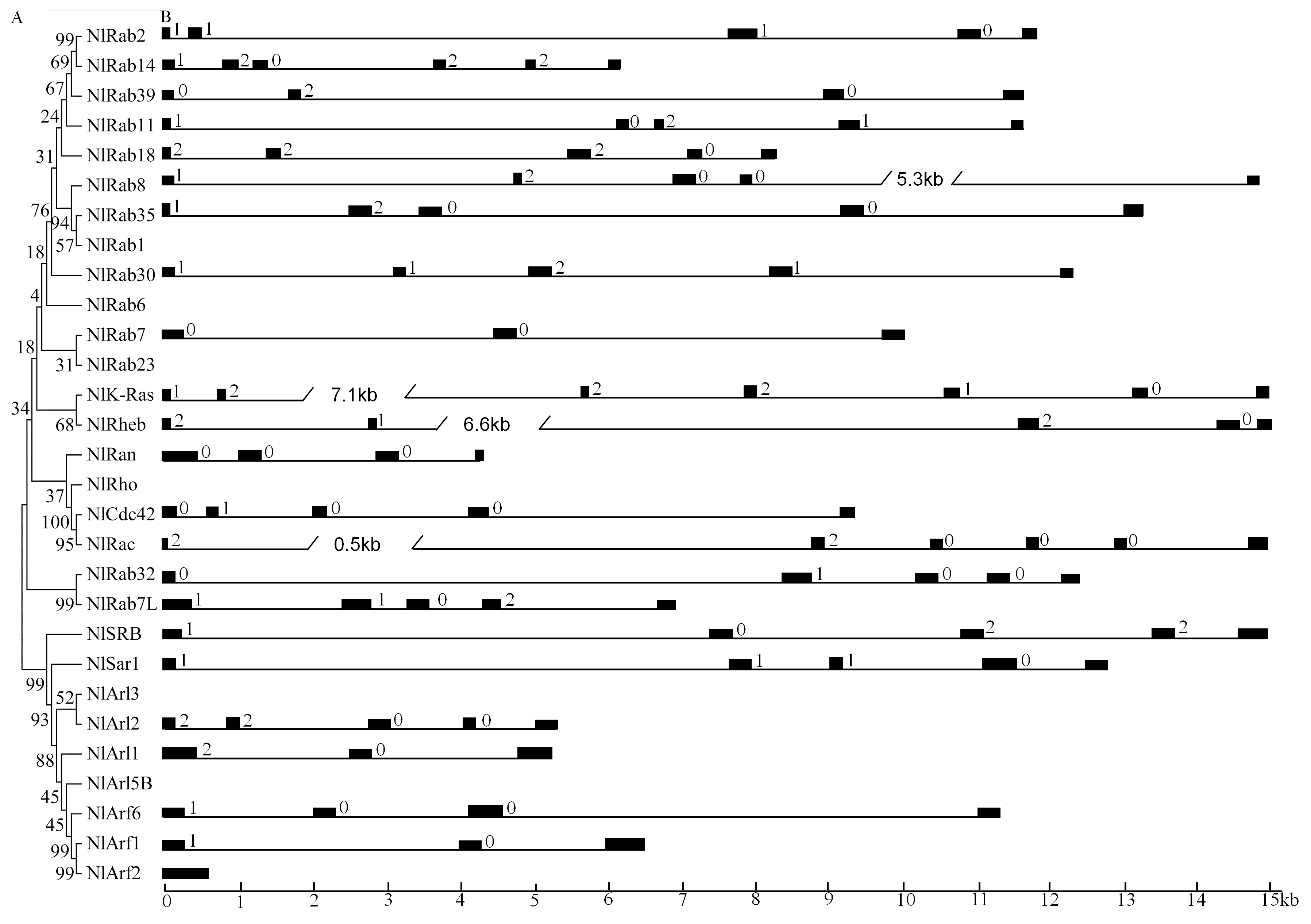

Supplement: S1 Fig — The number of branches on the phylogenetic tree indicated the bootstrap values. The phylogenetic tree was constructed by maximum likelihood based on the full-length cDNAs. cDNA and genomic sequences were compared putative exon–intron map. Exons and introns are indicated with the black boxes and black lines, respectively. The number indicated intron phase. Lengths are roughly at scale. (TIF) [file pone.0172701.s001.tif]

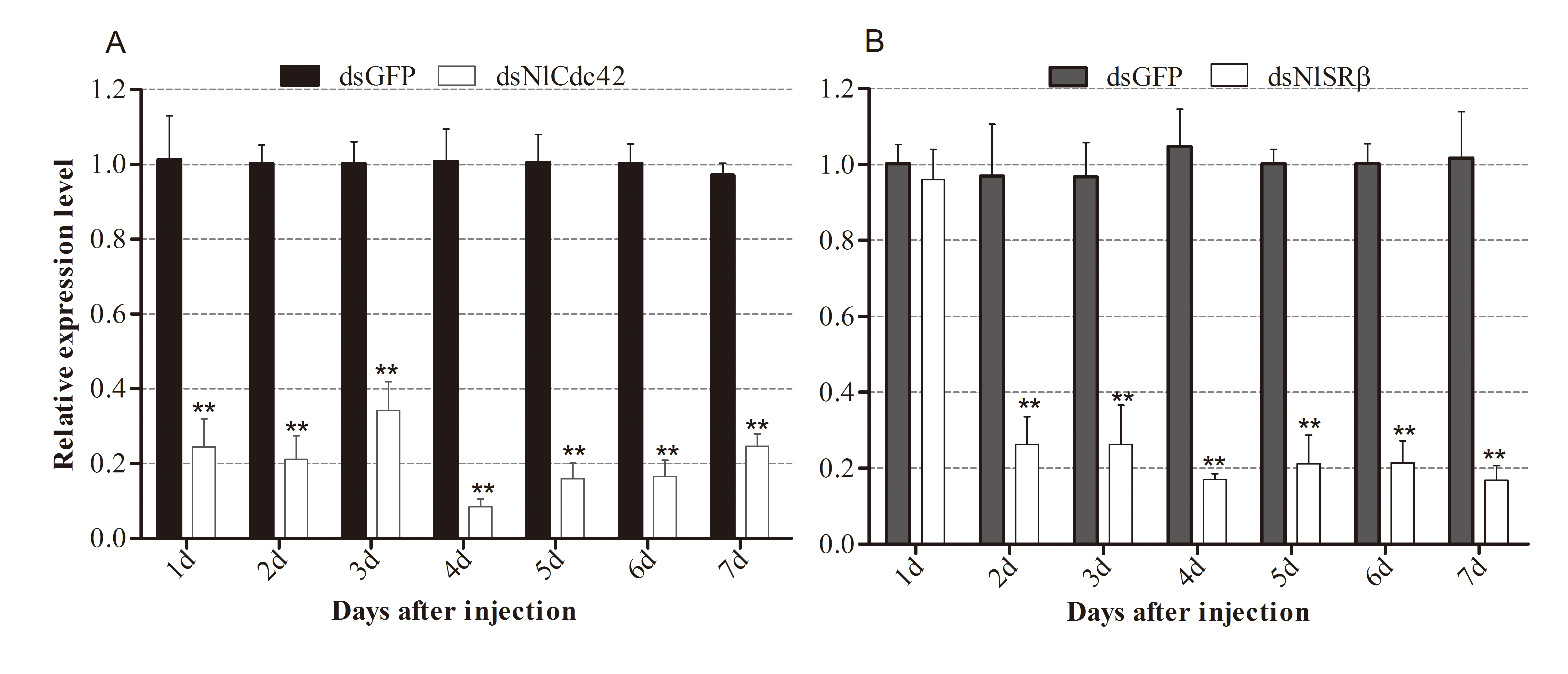

Supplement: S2 Fig — The relative transcript level for each sample was measured daily from 3 independent pools of 5 nymphs. P < 0.01 was considered statistically significant (**) different from dsGFP treatment. (TIF) [file pone.0172701.s002.tif]

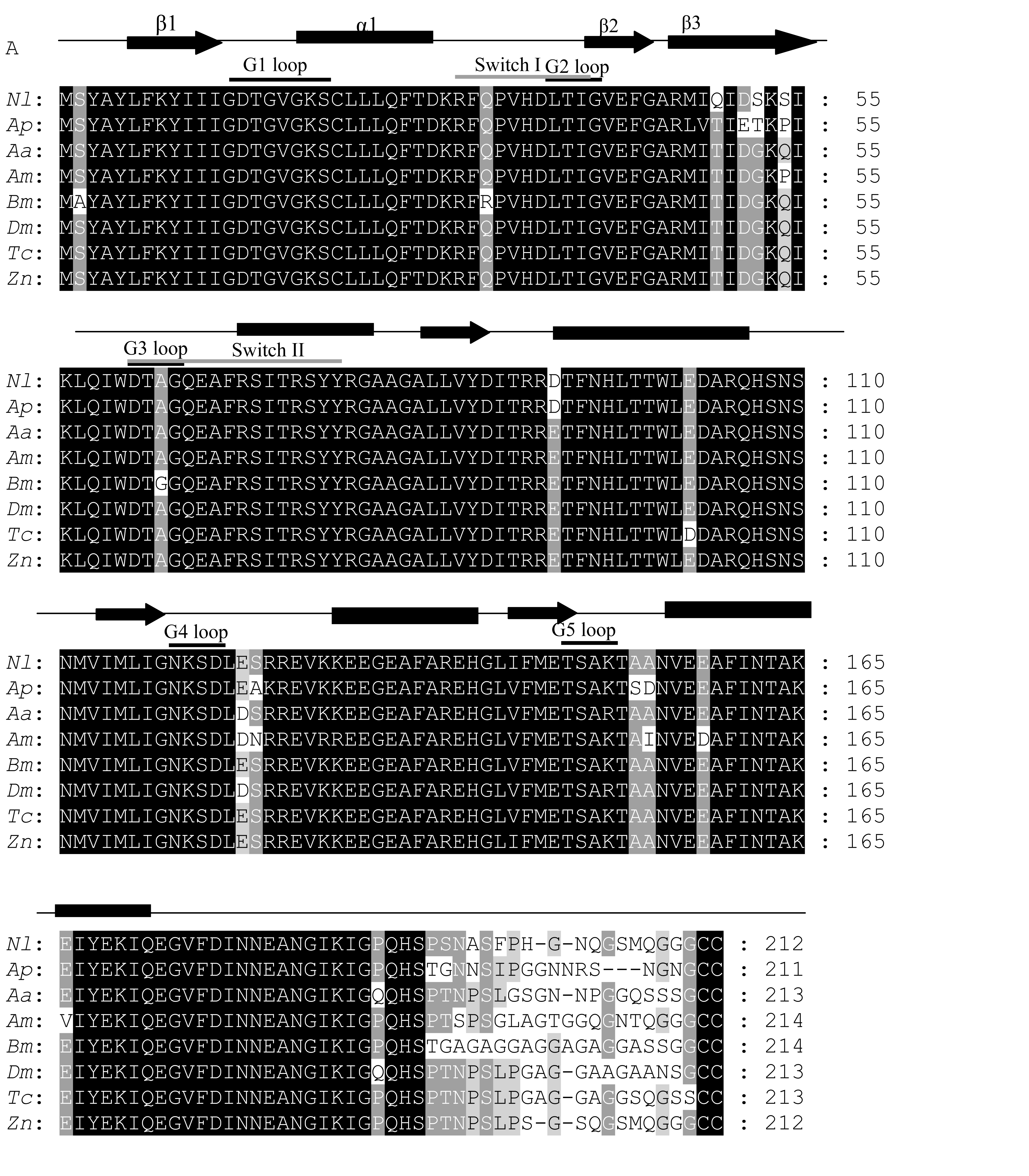

Supplement: S3 Fig — Blank residues indicate identical amino acids. Gray residues represent conserved substitutions. Sequences were aligned using ClustalW. Nl,N.lugens;Ap, Acyrthosiphon pisum;Bm, Bombyx mori;Zn, Zootermopsis nevadensis;Aa, Aedes aegypti;Dm, Drosophila melanogaster;Am, Apis mellifera;Tc, Tribolium castaneum. The data sources for all Ras family GTPase are listed in S3 Table. (TIF) [file pone.0172701.s003.tif]

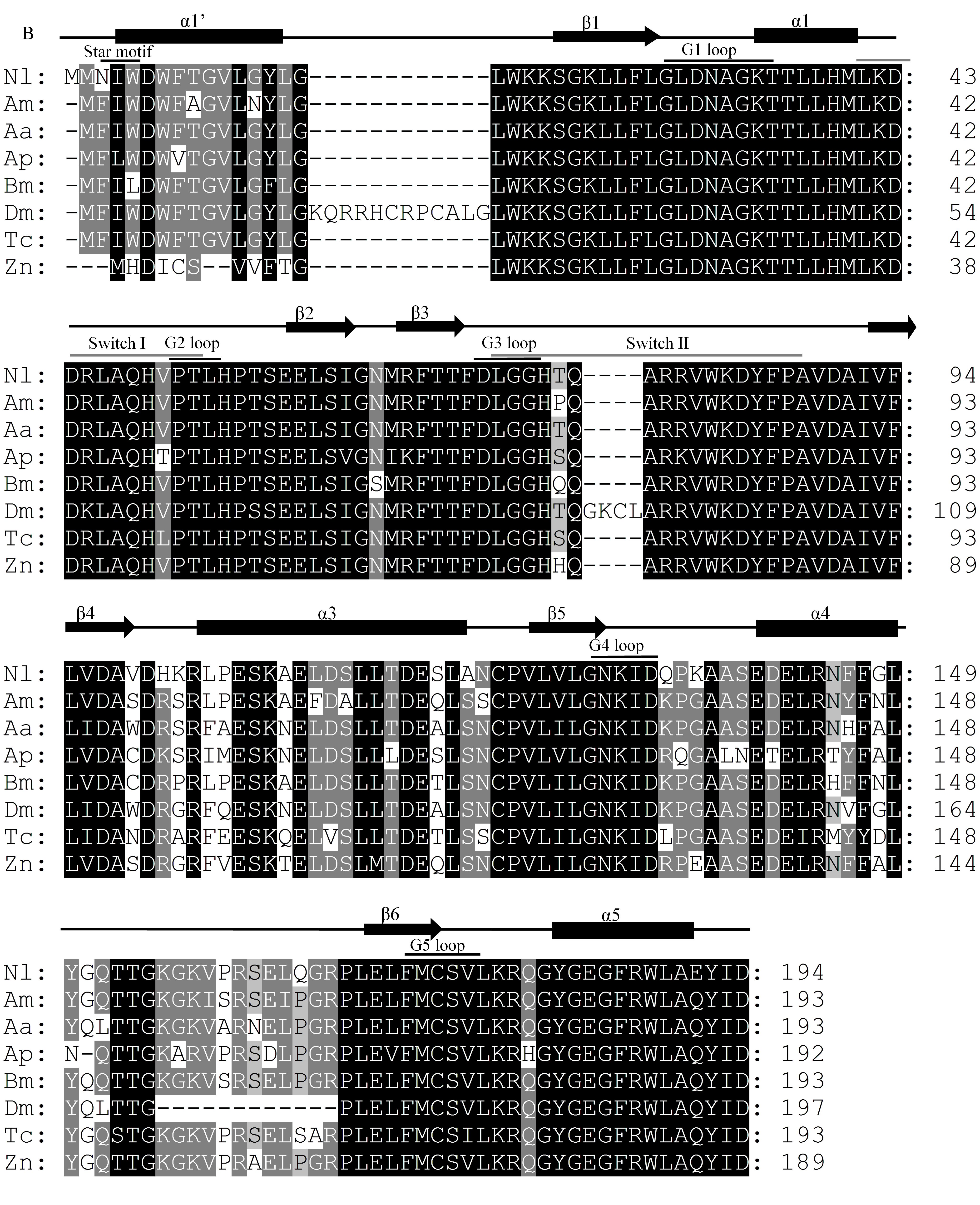

Supplement: S4 Fig — Blank residues indicate identical amino acids. Gray residues represent conserved substitutions. Sequences were aligned using ClustalW. Nl,N.lugens;Ap, Acyrthosiphon pisum;Bm, Bombyx mori;Zn, Zootermopsis nevadensis;Aa, Aedes aegypti;Dm, Drosophila melanogaster;Am, Apis mellifera;Tc, Tribolium castaneum. The data sources for all Ras family GTPase are listed in S3 Table. (TIF) [file pone.0172701.s004.tif]

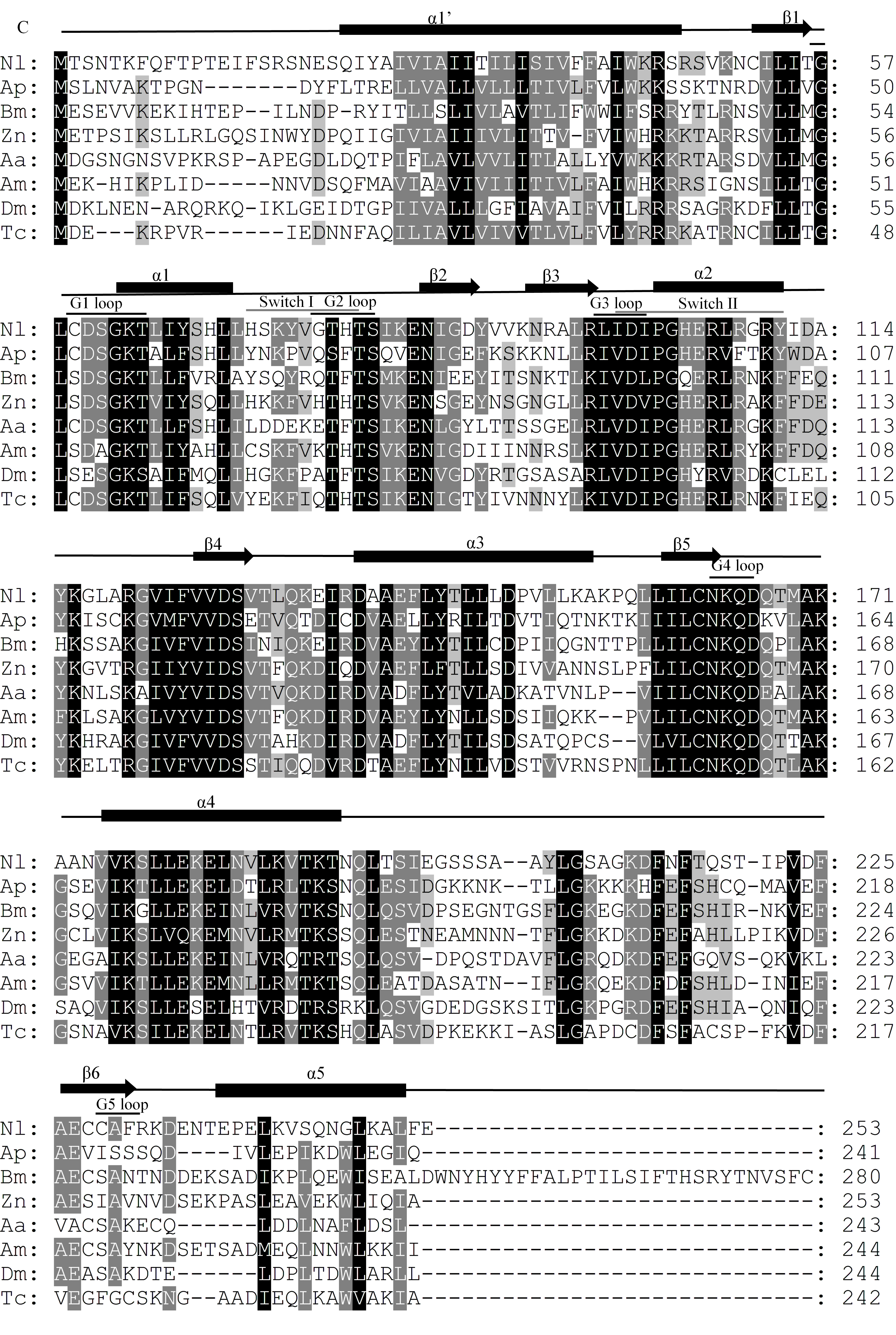

Supplement: S5 Fig — Blank residues indicate identical amino acids. Gray residues represent conserved substitutions. Sequences were aligned using ClustalW. Nl,N.lugens;Ap, Acyrthosiphon pisum;Bm, Bombyx mori;Zn, Zootermopsis nevadensis;Aa, Aedes aegypti;Dm, Drosophila melanogaster;Am, Apis mellifera;Tc, Tribolium castaneum. The data sources for all Ras family GTPase are listed in S3 Table. (TIF) [file pone.0172701.s005.tif]
