# Supplementary material for: Ras-like family small GTPases genes in Nilaparvata lugens: Identification, phylogenetic analysis, gene expression and function in nymphal development
Source: PLoS One. 2017 Feb 27;12(2):e0172701. doi: 10.1371/journal.pone.0172701 (PMC5328259; doi:10.1371/journal.pone.0172701)
Supplement: S1 Table — F,forward primer,R,reverse primer. (DOC) [file pone.0172701.s006.doc]

Supplementary Table 1

| **Primer name** | | **Sequence(5’ to 3’)** | **Amplicon size(bp)** |
| --- | --- | --- | --- |
| **Primer for clone** | | | |
| NlRab30 | F: AGCAATTAAGTGATGCAGAGTGAC  R: GGAGACACCTTGTAGTGGGGT | | 749 |
| NlRab35 | F: CGTGTAATTTTACCCCCAGCTTT  R: TTTCCATAAATGCAACCGAAGTCAT | | 709 |
| NlRab32 | F: GGCTTCACTTTGCACACGTT  R: ATGCCCCACACGATGTGAAT | | 898 |
| NlSar1 | F: ACAAGCGTTTCTGTCACTTTGTT  R: CACTAAATACGGAAAGTAGACACAG | | 1200 |
| NlRab18 | F: GGACAAGTGGATGGAGTAGAGG  R: CATAACAAAAACCCCGCGCC | | 950 |
| NlRab8 | F: GGGAAGTGACAAGTGACGGAA  R: TGATTGTTCAGCCATGCACC | | 1017 |
| NlRab2 | F:GGCTACTGCGACTCACTTATCT  R: CTGAGATGAGCGTCTACTCCC | | 791 |
| NlRab7 | F: AAAGCAGCAAGCACTCTAAGC  R: ATCCTAACCGTAGGAAGGGC | | 1018 |
| NlRab11 | F: AATGAGAGGGGCGTAGTGAAG  R:ACTCCATCCGACAAGCTCAC | | 918 |
| NlK-Ras | F:GTGGTGTGCATACTGTGCAAT  R: GGCAGTGATGAGTAGCCCAG | | 967 |
| NlRho | F:TGTGGCATTTCTCGCTGACG  R: TTGACTGGTTTGACAGTTTGGT | | 998 |
| NlRab23 | F:AGTGTCAGTTCCTTGCGACC  R:TCACGTTGTCGCTTCGGTTT | | 1179 |
| NlRab1 | F: AGACGGACGTTAAAGTGTTCC  R: TGTGGAGATGGACAGGGTCT | | 699 |
| NlRab14 | F:TACGGCCGGGGGTTGTCA  R: ACAAGTTTTGTGTCAAGGGT | | 1228 |
| NlArf1 | F：TACGACCGCCATCTTTTCCC  R： GCCGTTTACAACCGGTTTCC | | 898 |
| NlRab7L | F：GAAACGACAAGTGTGATGTCCG  R：AACACCTCGTTCTCTCGGGT | | 805 |
| NlCdc42 | F：TGATAAGTTTTGTTGACGCCC  R：TGGAGATGACTCTTGGGCTG | | 711 |
| NlArf6 | F：GTATGCGTTGACTCGCTGTC  R：CGAACACAAATGTGCGAACAAC | | 842 |
| NlArl3 | F：GTCCCGGCTACTTTGTCGT  R：ATTTGAATAATGGACATCTTGCCA | | 927 |
| NlArf2 | F：AATTTGGTGCTGTATGCGGT  R：GGCTTACAACTAGTAAACACCGA | | 830 |
| NlRheb | F：AATTGTGAACCTGGTGTTGTTT  R：GCAATCTTTGCGCTTCAACAC | | 989 |
| NlArl5B | F：TTGGCAACGAGGAGCACAAG  R：ATTTCGCACTCACACACTGAAA | | 797 |
| NlRab6 | F: TGATCCGTGGACGGGTGTAA  R: ATTTGTGAGCGTTGCAGTCTC | | 1201 |
| NlArl2 | F：TCTTTGTTCAAAAATGGGTCTGCT  R：GCAACCGGCACCTAGTCTTT | | 745 |
| NlRab39 | F: AGGACGTCATTCAAAACCTTCA  R: TGACGTCACTAATCCGTGTTCTT | | 875 |
| NlArl1 | F：ATCACCAGCACCAGTCACCA  R：TGTTCAGATGCAAGACGGCAA | | 795 |
| NlRac | F：TTAACCTCCCCACCCATTCA  R：ACGTCAGCTTCGAACATTGTC | | 1223 |
| NlSRβ | F:GCTGTCTCCCGTTTGGAAAAGT  R:AAAGCTTTCAAGCCATTTTGGGA | | 871 |
| **Primer for qRT-PCR** | | | |
| NlRab30 | F：CCTTTGACTGCCTGCCTGA  R：TGAGCTTCCTTGGCAGAAGT | | 181 |
| NlRab35 | F：GGATACCGCTGGACAAGAAAGA  R：GGGGGCGTCGTTTTTATTACC | | 190 |
| NlRab32 | F: TACAGAGCAACAATCGGCGT  R: CCGTCTGGAAGCTGGACTTT | | 239 |
| NlSar1 | F: GTCACACACAAGCGAGGAGA  R: CTTGGGCTGGTCGATCTTGT | | 188 |
| NlRab18 | F: CCAGTTACTACCGGGATGCG  R: ATCATCACGTGACACTTCCCT | | 182 |
| NlRab8 | F: CGGGGAGCAATGGGAATCAT  R: CGCCAATTGTTCGCCTCTTT | | 186 |
| NlRab2 | F：TTCAGGTCGATCACTCGCTC  R：GGCTGTGTTGATGAAAGCCTC | | 291 |
| NlRab7 | F: GCGCCGACTTCCTCACAAAA  R: GCTGGGCACGTTTCGTTG | | 297 |
| NlRab11 | F：GACACAGCCGGTCAAGAGAG  R：GCCGATGTCTCGATGAAGGA | | 269 |
| NlK-Ras | F：GTACAGTGCCATGAGAGACCA  R：ACCTTGCCTAGTTTTCGCTG | | 265 |
| NlRho | F: ACAGACTCCGCCCTCTATCA  R: GCCAACTCGCGGATGGTATT | | 196 |
| NlRab23 | F：TTCCACAATGCGGGAAGAGG  R：AGTGCCCTTGCAGTACCTT | | 106 |
| NlRab1 | F:ACTGCACCGACCAGGAGTCGT  R:GTCGCGTTCTTCGCCGACGT | | 202 |
| NlRab14 | F：GGTGTGGAATTTGGAACCAGG  R: ACGTCTCTCTTGTTGCCGAT | | 250 |
| NlArf1 | F: CGGCGGTCAGGACAAAATCA  R: GTTGGGCAGGTCCTGTTTGT | | 193 |
| NlRab7L | F：ACAACTATGGGATATTGCAGGACA  R：CACGGAATTGGGCTTCCATC | | 183 |
| NlCdc42 | F：CATCACTGTCAGAAAACGCCA  R：TTCTGGGTTAATGCGGAGCA | | 182 |
| NlArf6 | F：GCTGTGGCGACACTACTACA  R：GGCAGATCCTGTTTGTTGGC | | 165 |
| NlArl3 | F：TACGTGATAGACAGCGCCGA  R：GCCGGTCCTTGATCTGGTG | | 187 |
| NlArf2 | F：TGGGATGTCGGTGGTCAAAA  R：TCGGGAGATCCTGTTTATTCGC | | 199 |
| NlRheb | F：AAACTGGTTGATACGGCGGG  R：GCCAACAAGCACAATAGGCA | | 186 |
| NlArl5B | F：ACAAGCAGGACATCAACGGG  R：ACCTGCTCACGATCCATTCC | | 153 |
| NlRab6 | F: ACCGATTTGTCCGATAAGCGA  R: TGCATGTCTTCGGGTGGTTT | | 185 |
| NlArl2 | F：TGTGAAGATACGGGCAGCAA  R：AGCCACTCTGCTGGTGTTTG | | 196 |
| NlRab39 | F: AGTGAAAGATGGCGCAAGGA  R: GAAGCTGGCTCGGTTGCATA | | 139 |
| NlArl1 | F：ACAATTGGATTCAACGTGGAGC  R：ATCTGCCGAGTCCACAACAT | | 147 |
| NlRac | F：CGACCACTGTCTTACCCACA  R：TGTTTCTTTGTCCTCGCGCA | | 174 |
| NlSRβ | F:AGCACGTGGAGTGATCTTTG  R:TTCCTGCGCTCCCAAGATAG | | 281 |
| **Primer for dsRNA** | | | |
| NlRab30 | F: AGCAATTAAGTGATGCAGAGTGAC  R: GGAGACACCTTGTAGTGGGGT | | 749 |
| NlRab35 | F:CCCAGCTTTATCAATTCATCTGCC  R: TTTCCATAAATGCAACCGAAGTCA | | 695 |
| NlRab32 | F:GGCTTCACTTTGCACACGTT  R: ATGCCCCACACGATGTGAAT | | 898 |
| NlSar1 | F:CACACACAAGCGAGGAGAGT  R: ACAACGCTGAAGAACTGCCT | | 568 |
| NlRab18 | F:GACAAGTGGATGGAGTAGAGGA  R: TCCCGGTAGTAACTGGGTGT | | 515 |
| NlRab8 | F:CGGGTCAGGAAAGGTTCAGG  R: GTTGACCGCGTCGACTCTAA | | 639 |
| NlRab2 | F:GCTCCAGTTTACAGACAAGAGGT  R: TCCCTCCTGGATCTTCTCGT | | 454 |
| NlRab7 | F:TGACACTGGAGTTGGCAAAACA  R: GGTGAGCTTGATCTGGTCTGG | | 535 |
| NlRab11 | F:CCGTGATGACGAATATGACTAT  R: CTGGCAACACTGTTTGCGAA | | 637 |
| NlK-Ras | F:CGCACCTGAAGTATGACGGA  R: TGCTGTTGGCCACGAGTTAG | | 595 |
| NlRho | F:TTATCGTCGGTGATGGTGCTT  R: ACACCTGACGAACTCCTTCC | | 486 |
| NlRab23 | F:GTCAGCGTGAGTGTAACGGA  R: TTGACAACAGATCGGCCTCC | | 600 |
| NlRab1 | F:AGACGGACGTTAAAGTGTTCC  R: TGTGGAGATGGACAGGGTCT | | 699 |
| NlRab14 | F:TATCATCATTGGTGATATGGGC  R: ACGTCTCTCTTGTTGCCGAT | | 342 |
| NlArf1 | F:ACGCAGCTGGTAAAACCACA  R: TCCAACAACCAGTTGGGAGG | | 662 |
| NlRab7L | F:GCCAGTGACCCAGAAAGTGA  R: ACCTCGTTCTCTCGGGTGTA | | 664 |
| NlCdc42 | F:AATGCGTTGTCGTGGGAGAT  R: TGGAGATGACTCTTGGGCTG | | 657 |
| NlArf6 | F:TTGGCGAGCTTTGATCCACA  R: CAGTGTCGGCTCTAGTGGTG | | 598 |
| NlArl3 | F: CCGGCTACTTTGTCGTGCAT  R: TTCCATCCTTGATGCCCTCG | | 608 |
| NlArf2 | F: ATTTGGTGCTGTATGCGGTG  R: TCGCACATGTTGCTTGGATG | | 646 |
| NlRheb | F: GAAACTGGTTGATACGGCGG  R: ACCCACTTTGGTTATGAAACATCC | | 520 |
| NlArl5B | F: GTCGAGGAAGTCGTCTGGAA  R: TTTCGCACTCACACACTGAAA | | 677 |
| NlRab6 | F: GTGAGGCTTCAGTTGTGGGA  R: GTTTGTTGCACCACAACCCT | | 605 |
| NlArl2 | F: CAACCGGCACCTAGTCTTTTC  R: TTACAAGCTGAACGTGTGGGA | | 549 |
| NlRab39 | F: AGTGCTTCATAATGGTGGACCC  R: ACCTTGTATTCGCCCGACTG | | 571 |
| NlArl1 | F: TGAGCCTTGCCTTGTCTACG  R:AAGCCCTCTCCTTTGACAGC | | 614 |
| NlRac | F: GCAAGCCATAAAGTGTGTGGT  R: TGACGCATTTCCGTCGGT | | 569 |
| NlSRβ | F: ATCTCCATTGTATTCTTCGC  R: TTCCTGCGCTCCCAAGATAG | | 532 |
