# Supplementary material for: Ras-like family small GTPases genes in Nilaparvata lugens: Identification, phylogenetic analysis, gene expression and function in nymphal development
Source: PLoS One. 2017 Feb 27;12(2):e0172701. doi: 10.1371/journal.pone.0172701 (PMC5328259; doi:10.1371/journal.pone.0172701)
Supplement: S2 Table — (DOCX) [file pone.0172701.s007.docx]

**Supplementary Table 2. Protein sequences sources used in Fig 1**

|  | Systematic Name | Accession No. | Length aa |
| --- | --- | --- | --- |
| 1 | ZnRab1 | KDR20725.1 | 206 |
| 2 | TcRab1 | XP_975150.1 | 202 |
| 3 | ZnRab2 | KDR09308.1 | 213 |
| 4 | TcRab2 | XP_971919.1 | 214 |
| 5 | ZnRab6 | KDR24391 | 210 |
| 6 | TcRab6 | XP_008200265 | 209 |
| 7 | ZnRab7 | KDQ65243.1 | 206 |
| 8 | AmRab7 | XP_001120025.1 | 207 |
| 9 | ZnRab8A | KDR18832 | 177 |
| 10 | TcRab8 | XP_015836858.1 | 209 |
| 11 | ZnRab11 | KDR19906.1 | 215 |
| 12 | DmRab11 | NP_477170.1 | 214 |
| 13 | ZnRab14 | KDR15105.1 | 215 |
| 14 | TcRab14 | NP_001153783.1 | 215 |
| 15 | ZnRab18 | KDR14057.1 | 206 |
| 16 | TcRab18 | XP_970378.1 | 204 |
| 17 | ZnRab23 | KDR12233.1 | 236 |
| 18 | TcRab23 | XP_975612.1 | 232 |
| 19 | ZnRab30 | KDR16816.1 | 203 |
| 20 | TcRab30 | XP_970787.2 | 203 |
| 21 | AmRab32 | XP_016772353.1 | 265 |
| 22 | BmRab32 | XP_012551072.1 | 236 |
| 23 | ZnRab35 | KDR22454.1 | 237 |
| 24 | TcRab35 | XP_967046.1 | 201 |
| 25 | TcRab39 | XP_969295 | 218 |
| 26 | ZnRab39 | KDR14202.1 | 217 |
| 27 | ZnRab7L | KDR22884.1 | 260 |
| 28 | ApRab7L | XP_001951808 | 201 |
| 29 | ZnK-Ras | KDR07913.1 | 191 |
| 30 | ApK-Ras | NP_001155401.1 | 189 |
| 31 | ApRheb | XP_008179467.1 | 193 |
| 32 | ZnRheb | KDR11610.1 | 182 |
| 33 | ZnRho | KDR13982.1 | 192 |
| 34 | ApRho | NP_001155513.1 | 192 |
| 35 | ZnCdc42 | KDR11574.1 | 191 |
| 36 | TcCdc42 | XP_966688.1 | 191 |
| 37 | TcRac | XP_968397 | 192 |
| 38 | ApRac | XP_001943865 | 192 |
| 39 | ZnRan | KDR15447 | 215 |
| 40 | TcRan | XP_966512 | 215 |
| 41 | ZnSar1 | KDR11939.1 | 190 |
| 42 | TcSar1 | XP_968802.1 | 194 |
| 43 | ZnSRβ | KDR07565.1 | 254 |
| 44 | TcSRβ | XP_971825.1 | 243 |
| 45 | ApArf1 | XP_001944342.1 | 182 |
| 46 | TcArf1 | XP_968387.1 | 182 |
| 47 | ZnArf2 | KDR19101.1 | 175 |
| 48 | TcArf2 | XP_001808435.1 | 180 |
| 49 | ZnArf6 | KDR09315.1 | 215 |
| 50 | TcArf6 | XP_967713.1 | 175 |
| 51 | ZnArl1 | KDR23846.1 | 180 |
| 52 | TcArl1 | XP_973025.1 | 180 |
| 53 | ZnArl2 | KDR20929.1 | 184 |
| 54 | TcArl2 | XP_975625.1 | 184 |
| 55 | ZnArl3 | KDR16095.1 | 218 |
| 56 | ApArl3 | XP_001949993.1 | 180 |
| 57 | ZnArl5B | KDR11571.1 | 179 |
| 58 | TcArl5A | XP_971281.1 | 179 |

Ap, *Acyrthosiphon pisum*;Bm, *Bombyx mori*;Zn, *Zootermopsis nevadensis*; Am, *Apis mellifera*;Tc, *Tribolium castaneum*.
