# Supplementary material for: Ras-like family small GTPases genes in Nilaparvata lugens: Identification, phylogenetic analysis, gene expression and function in nymphal development
Source: PLoS One. 2017 Feb 27;12(2):e0172701. doi: 10.1371/journal.pone.0172701 (PMC5328259; doi:10.1371/journal.pone.0172701)
Supplement: S3 Table — (DOC) [file pone.0172701.s008.doc]

**Supplementary Table S3. Sequences sources used in Table 2 and Supplementary Fig S1**

|  | Systematic  Name | Protein  Accession No. | cDNA  Accession No. | Length of  Protein(aa)/ORF(bp) |
| --- | --- | --- | --- | --- |
| 1 | BmSar1 | XP_004931167.1 | XM_004931110.2 | 194/582 |
| 2 | ApSar1 | XP_008187339.1 | XM_008189117.1 | 193/579 |
| 3 | TcSar1 | XP_968802.1 | XM_963709.4 | 194/582 |
| 4 | AmSar1 | XP_006563700.1 | XM_006563637.1 | 194/582 |
| 5 | DmSar1 | CAB81550.1 | AJ276483 | 198/594 |
| 6 | AaSar1 | ABF18297.1 | DQ440264.1 | 194/582 |
| 7 | ZnSar1 | KDR11939.1 | KK853056.1 | 190/570 |
| 8 | ZnRab2 | KDR09308 | KK853253.1 | 213/639 |
| 9 | AaRab2 | XP_001650005.1 | XM_001649955 | 214/642 |
| 10 | TcRab2 | XP_971919.1 | XM_966826 | 214/642 |
| 11 | DmRab2 | NP_477090.1 | NM_057742.4 | 214/642 |
| 12 | BmRab2 | NP_001037612.1 | NM_001044147 | 215/645 |
| 13 | ApRab2 | XP_008183935.1 | XM_008185713.2 | 212/636 |
| 14 | AmRab2 | XP_392651.2 | XM_392651 | 215/645 |
| 15 | ApSRβ | XP_001944151.1 | XM_001944116.4 | 242/726 |
| 16 | TcSRβ | XP_971825.1 | XM_966732.3 | 243/729 |
| 17 | AmSRβ | XP_393949.2 | XM_393949.5 | 245/735 |
| 18 | BmSRβ | NP_001091817.1 | NM_001098347.1 | 281/843 |
| 19 | DmSRβ | NP_788485.1 | NM_176307.3 | 245/735 |
| 20 | AaSRβ | EAT38257.1 | CH477616.1 | 244/732 |
| 21 | ZnSRβ | KDR07565.1 | KK853448.1 | 254/762 |

Ap, *Acyrthosiphon pisum*;Bm, *Bombyx mori*;Zn, *Zootermopsis nevadensis*;Aa, *Aedes aegypti*;Dm, *Drosophila melanogaster*;Am, *Apis mellifera*;Tc, *Tribolium castaneum*.
